# Exosome-mediated modulation of radioresistance: The radiation-induced bystander effect in prostate cancer cells

**DOI:** 10.1371/journal.pone.0330501

**Published:** 2025-08-28

**Authors:** Beata Pszczółkowska–Kępa, Wioletta Olejarz, Alicja Głuszko, Grzegorz Wałpuski, Tomasz Lorenc, Beata Brzozowska

**Affiliations:** 1 Biomedical Physics Division, Faculty of Physics, University of Warsaw, Warsaw, Poland; 2 Department of Biochemistry and Pharmacogenomics, Faculty of Pharmacy, Medical University of Warsaw, Warsaw, Poland; 3 Centre for Preclinical Research, Medical University of Warsaw, Warsaw, Poland; 4 Chair and Department of Biochemistry, Medical University of Warsaw, Warsaw, Poland; 5 Department of Molecular Plant Physiology, Faculty of Biology, University of Warsaw, Warsaw, Poland; 6 Department of Radiology I, The Maria Sklodowska-Curie National Research Institute of Oncology, Warsaw, Poland; University of Mississippi Medical Center, UNITED STATES OF AMERICA

## Abstract

Exosomes are involved in intracellular communication and mediate the radiation-induced bystander effect (RIBE). We assessed the ability of exosomes to modify the radiation response of PC3 and DU145 prostate cancer cells exposed to X-rays. Irradiated cells were analyzed using clonogenic survival and apoptosis assays, while exosome-stimulated cells were evaluated for *γ*H2AX immunostaining, immunoblotting, and apoptosis. Exosomes were isolated via size exclusion chromatography (SEC), characterized by nanoparticle tracking analysis (NTA) and immunoblotting, and ranged from 130 to 137 nm, containing CD63 and CD81. We found that exposure to ionizing radiation (IR) resulted in increased apoptosis and necrosis. To assess exosomes impact on radiation response, exosomes were transferred to non-irradiated and irradiated recipient cells. Non-irradiated PC3 cells stimulated by exosomes released from irradiated PC3 and DU145 cells showed more apoptosis and necrosis than those stimulated by exosomes released from non-irradiated cells. Non-irradiated PC3 cells co-incubated with exosomes from irradiated PC3 and DU145 cells exhibited more *γ*H2AX foci than non-irradiated PC3 cells. Our results confirmed that DU145 cells are more radioresistant than PC3 cells and exosomes isolated from these cells may contribute to radiation resistance in prostate cancer. Thus, studying exosome functions, particularly in radiation resistance, is crucial for understanding carcinogenesis and optimizing radiotherapeutic methods.

## Introduction

Radiotherapy (RT) is one of the cornerstone modalities for cancer treatment, with over 60% of cancers managed either solely through radiation or in combination with chemotherapy or surgery. As a predominantly local treatment option, radiotherapy can be used with either palliative or curative intent, particularly in cases of oligometastatic or oligoprogressive disease, where stereotactic body radiation therapy (SBRT) has shown efficacy [1,2]. Traditionally, radiobiological effects of ionizing radiation were attributed to direct DNA damage (e.g., single- and double-strand breaks, DNA-DNA and DNA-protein crosslinks, and chromosomal aberrations) or indirect damage via reactive oxygen species (ROS) generated through water radiolysis. These processes, collectively described as radiation-induced targeted effects, occur in irradiated cells as a direct result of energy deposition [3].

However, this conventional paradigm has evolved. Emerging evidence suggests that radiation effects extend beyond the irradiated cells, influencing non-irradiated neighboring or distant cells through mechanisms such as genetic mutations, chromosomal damage, micronucleus formation, and apoptosis [4–6]. These phenomena, collectively termed radiation-induced non-targeted effects, are mediated by intercellular communication and the release of molecular signals or extracellular vesicles by irradiated cells. Such systemic effects have even been linked to carcinogenic outcomes outside the radiation field [7,8].

Exosomes are a subset of extracellular vesicles (EVs), which are secreted by cells, with a distinct size range of 30 to 150 nm in diameter, formed through inward budding of the endosomal membrane [9]. In contrast, small EVs (sEVs) encompass a broader range of vesicles, typically less than 200 nm in diameter, which includes exosomes as well as other vesicle types with different biogenetic origins [10]. The molecular cargo within exosomes, including proteins, lipids, and nucleic acids, reflects the state of the donor cell [11]. Exosomes play a pivotal role in modulating the systemic response to radiation, including the bystander effect. Ionizing radiation is known to influence both the release and uptake of exosomes, as well as their molecular cargo. These vesicles contain a diverse array of biomolecules, such as RNA, DNA, and proteins, whose composition can be altered following radiation exposure of the parental cells. Several studies have implicated exosomal RNA in propagating radiation-induced non-targeted effects, while the roles of exosomal DNA and proteins in these processes are increasingly being recognized [12,13]. For example, exosomes from irradiated cells have been linked to the radiation-induced bystander effect (RIBE), with both harmful and protective effects on target cells [14]. Exosomes are involved in intracellular communication and mediate the radiation-induced bystander effect through miRNA delivery (e.g., miR-21, miR-1246) [15], DNA cargo that activates innate immune sensors (e.g., cGAS-STING) [16], and surface proteins that trigger receptor-mediated responses (e.g., FasL) [17]. In breast cancer models, exosomes caused chromosomal damage and genetic instability [18–21]. However, those from irradiated head and neck cancer cells were shown to enhance DNA repair and survival in recipient cells [22]. In studies on human non-small cell lung cancer cell lines (H460, H1299) as donor cells, RIBE in recipient cells after 24 hour of incubation was shown to induce a p53-dependent response to DNA damage, indicating that the p53 pathway regulates exosome production in cancer cell communication [23]. These findings underscore the complex, context-dependent nature of exosome involvement, although the underlying molecular mechanisms remain incompletely understood.

Prostate cancer remains a primary indication for radiotherapy and is widely considered as a standard of care for this malignancy [24]. Nonetheless, the therapeutic efficacy of radiation is influenced by the intrinsic radiosensitivity of the tumor cells, which varies among different cell populations within the tumor [25]. EVs hold promise for cancer progression monitoring, biomarker discovery, and enhancing prostate cancer radiosensitivity [26,27]. To date, there is a limited literature examining the role of exosomes in mediating the bystander effect following irradiation of prostate cancer cells with varying radiosensitivity. This gap in knowledge serves as the motivation for the present study. Here, we employed PC3 and DU145 cell lines, representative models of human prostate cancer, to observe exosome-mediated radiation-induced bystander effects. These cell lines were selected for their distinct radiosensitivity profiles. The study analyzed the effects of two radiation doses delivered with X-rays with differential impacts on cell survival to provide a comprehensive understanding of radiation-induced bystander phenomena. The primary objective of this work was to investigate the role of exosomes in mediating radiation-induced effects in prostate cancer cells using the survival and apoptotic studies. It was already demonstrated that cancer cells can exhibit resistance to radiotherapy or chemotherapy by disrupting the signaling pathways that induce apoptosis [28]. Additionally, the DNA damage analysis is included based on the microscopic images of *γ*H2AX, which is a widely accepted marker of DNA strand breaks that shows a dose-dependence for radiation-induced DNA double-strand breaks [29].

## Materials and methods

### Cell culture and irradiation

PC3 (CRL-1435) and DU145 (HTB-81) cell lines were purchased from American Cell Type Collection (ATCC). Cells were cultured in Dulbecco’s modified Eagle medium (DMEM - F12, VWR; 392-0411) supplemented with 10% foetal bovine serum (FBS, biowest; S1810-500) and 2.0% penicillin-streptomycin (Thermo Fisher Scientific, Gibco; 15140122). The medium was changed three times per week, and cells were incubated at $37^{\text{o}}\text{C}$, in a 5% ${\text{CO}}_{2}$ atmosphere. For all RIBE experiments and for isolation of sEVs, the standard culture medium was replaced with a fresh medium containing 5% Gibco Exosome-Depleted FBS (Thermo Fisher Scientific, Gibco; A2720801) to eliminate background extracellular vesicles and confirm that the observed effects were specifically induced by the exosomes isolated from the human prostate cancer cell lines. Cells were irradiated with X-rays at different doses depending on the assay: 0 Gy (control), 2 Gy and 6 Gy for apoptosis assay and NTA analysis; 0 Gy, 0.5 Gy, 1 Gy, 2 Gy, 4 Gy, 6 Gy and 8 Gy for clonogenic survival assay; and 0 Gy (control) and 2 Gy for *γ*H2AX immunostaining. Irradiation was performed using an X-ray tube (Philips PW 2273/20) installed at the Biomedical Physics Division, Faculty of Physics, University of Warsaw, for a tube voltage of 50 kV and a tube current of 10 mA. The dose rate was 3.4 Gy/min as determined with Gafchromic^™^ EBT4 films (Ashland Inc., Bridgewater, NJ; 973857).

### Isolation of sEVs

For vesicle isolation, PC3 and DU145 cells were grown in T175 flasks (GenoPlast Biotech S.A., GoogLab; G77090033) with 20 mL of medium for Western Blot analysis (two T175 flasks per one sample for isolation). For apoptosis assay and *γ*H2AX immunostaining cells were cultured in two glass coverslips (2.0 × 10^5^ cells were seeded per one glass coverslips) placed in the 9.3 cm Petri dish with 18 mL of medium. After 96 hours the cell supernatant was collected by decanting. To remove cellular debris, the supernatant underwent sequential centrifugation at room temperature (RT) for 10 minutes at 2000 × g, followed by 30 minutes at 10,000 × g at $4^{\text{o}}\text{C}$. The supernatant was then filtered with a 0.22 μm filter (Millex, MilliporeSigma; SLGP033RS) to remove large EVs, including potential apoptotic bodies or microvesicles. The filtered supernatant was concentrated to 1 mL using Vivacell100 concentrators (100,000 MWCO, Sartorius; VS2041). Next, the 1 mL of the concentrate was placed onto an Econo-Pac Chromatography Column (Bio-Rad; 7321010) filled with 10 mL of Sepharose CL-2B (Cytiva; GE17-0140-01). sEVs were eluted in 1 mL fractions using Dulbecco’s Phosphate Buffered Saline (PBS, Thermo Fisher, Gibco; 14190136), and fraction #4 was collected for downstream applications, as previously described [30].

### Nanoparticle tracking analysis (NTA)

The size and concentration of extracellular vesicles were determined using the ZetaView, equipped with the NTA analytical software (version 2.3, Particle Metrix GmbH, Inning am Ammersee, Germany). For each sample of both prostate cancer cell lines, three independent biological experiments, each with two technical replicates, were analyzed.

### Clonogenic survival assay

After irradiation, for survival determination, a clonogenic survival assay was performed to determine survival fraction of PC3 and DU145 cells exposed to X-rays doses ranging from 0.5 Gy to 8 Gy. PC3 and DU145 cells were trypsinized, counted, and plated in Petri dishes containing 15 mL of medium. For the control group, PC3 cells were seeded at a density of 9,000 cells per dish, while DU145 cells were seeded at 6,000 cells per dish. In the irradiated samples, seeding densities ranged from 11,000 to 40,000 cells/dish for PC3 and from 8,000 to 20,000 cells/dish for DU145, depending on the radiation dose. After plating, the cells were incubated for 14 days to allow colony formation. Colonies were then stained with Giemsa solution and quantified using countPHICS software [31]. The plating efficiency (*PE*) was determined by dividing the number of colonies formed by the number of cells seeded. Surviving fractions (*SF*) of irradiated cells were calculated as a ratio of the *PE* of irradiated cells and the *PE* of control cells kept at the same conditions as irradiated cells. Each dose was tested using three replicates within a single experiment, and the entire experiment was repeated independently three times. The dose-response relationship was fitted using the linear-quadratic (*LQ*) model, where *α* and *β* parameters are constant for a given cell line and *D* is the absorbed dose. The slope of the curve, described as the *α*/*β* ratio (in Gy), reflects the radiosensitivity of cells. Cells with high *α*/*β* ratios exhibit a relatively constant rate of cell killing with increasing dose, while those with a low *α*/*β* ratio display more pronounced curvature. This curvature corresponds to the two contributions to cell killing. The *α* parameter reflects death from “single-hit” events, such as lethal damage caused by high linear energy transfer (LET) radiation, while the *β* parameter accounts for “multiple-hit” events, typically associated with low LET radiation.

### Apoptosis assay

**Fig 1:**
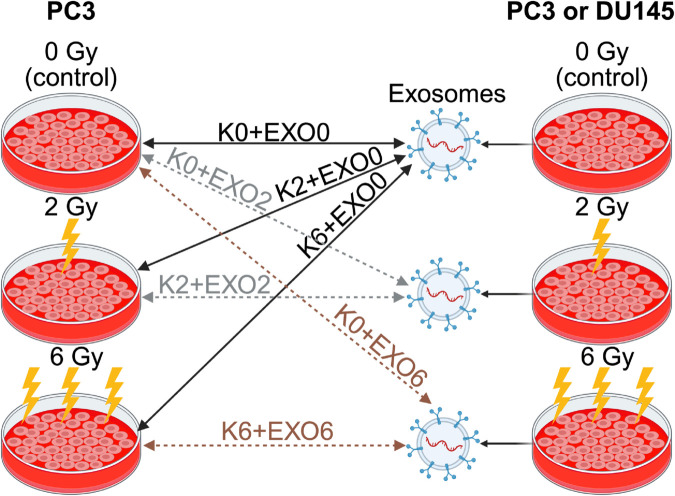
Schematic representation of the experimental groups applied in the RIBE apoptosis assay, illustrating the combination of exosome (EXO) sources, irradiation doses, and co-incubated PC3 recipient cell (K) conditions according to the Kx+EXOy notation.

Two days prior to irradiation, cells were seeded onto glass coverslips which were then placed in a 6-well plate. Cells were seeded at a density of 2.0 × 10^5^ cells per well. Following irradiation, the glass coverslips were transferred to Petri dishes and incubated for 96 hours. Cell apoptosis was assessed using the FITC:Annexin V Apoptosis Detection Kit I (BD Biosciences Pharmingen), following the manufacturer’s protocol. The cells were analyzed (10 000 cells per sample) with a FACSCalibur flow cytometer (Becton Dickinson) using CellQuest Software. For RIBE experiments, exosomes previously isolated were administered during the irradiation of the cells or to the control cells (Fig 1), which were prepared according to the scheme described above.

### Western blotting

For Western blot analysis, cells were seeded at a density of 2.6 × 10^5^ cells per 3.5 cm Petri dish, with three Petri dishes per sample. For RIBE experiments, cells were seeded onto three glass coverslip placed in a 9.3 cm Petri dish. The cells were incubated with exosomes isolated beforehand for 42 hours. Protein concentrations of cells and cells co-incubated with sEVs samples were measured using the Bradford protein assay [32]. Protein samples (20 μg) were solubilized in denaturing buffer (0.25 M Tris–HCl (pH 6.8), 0.4% SDS, 10 M urea, 2% 2-mercaptoethanol and 20% glycerol) and mixed together in 1:1 (v/v) ratio. The samples were then denatured at $75^{\text{o}}\text{C}$ for 10 minutes, and separated by 12% SDS-polyacrylamide gel electrophoresis. Proteins were subsequently transferred using the wet transfer method onto polyvinylidene fluoride (PVDF) membranes (Merck, IPVH00010). Incubation with primary antibodies anti-CD63 (1:1500, Thermo Fisher Scientific, Invitrogen; 10628D), anti-CD81 (1:500, Thermo Fisher Scientific, Invitrogen; 10630D), and Calnexin (1:1000, Thermo Fisher Scientific, Invitrogen; MA3-027) was performed overnight at $4^{\text{o}}\text{C}$, followed by incubation with secondary HRP-conjugated antibody (1:5000, Agrisera Antibodies, AS11 1772) for 1 h at RT. Visualization was performed by enhanced chemiluminescence method according to standard procedures using ChemiDoc System (Bio-Rad, USA).

### *γ*H2AX immunostaining

To enhance the visualization of *γ*H2AX foci, 10^5^ cells were seeded onto glass coverslips (22 mm × 22 mm, CHEMLAND; 298.202.08), which were then placed in 35 mm dishes for two days. PC3 prostate cancer cells were used as control (non-irradiated) cells. Exosomes were isolated from PC3 and DU145 cells, which were previously irradiated with 2 Gy of X-rays. In the next step, isolated exosomes were added to non-irradiated PC3 cells, followed by incubation periods of 1 and 3 hours. After incubation, cells co-incubated with sEVs were washed three times with PBS and fixed with 70% cold ethanol for 15 minutes at RT. After three washes with PBS, cells were permeabilized in 0.2% Triton X-100 for 5 min at RT and washed three times with PBS. Non-specific antibody binding was blocked by incubating cells with 3% BSA solution in PBS for 30 minutes at RT, after which cells were washed three times with PBS. The preparations were incubated with the primary antibodies anti-P-Ser139-H2A.X (1:400, Cell Signaling Technology; 9718S) in 3% BSA/PBS for 45 minutes at $37{\;}^{\text{o}}\text{C}$, followed by three PBS washes. Afterward, cells were incubated with a secondary antibody conjugated with FITC (1:1000, Abcam; ab67147-1) in 2% BSA/PBS for 45 min at $37{\;}^{\text{o}}\text{C}$, and nuclei were counterstained with VECTASHIELD^®^ PLUS Antifade Mounting Medium with DAPI (BIOKOM; H-2000-10). The *γ*H2AX focus induction by exosomes was visualized using an inverted optical microscope Olympus IX83 (Cellvivo, CellSens software) at a magnification of 100×.

### Statistical analysis

For the clonogenic survival and all apoptosis assays, three independent biological experiments were conducted, each including at least two technical replicates to ensure reliability and reproducibility of the data. All statistical analyses were performed using Python 3 (version Python 3.10.12, Python Software Foundation) with additional libraries, including SciPy and Matplotlib. For statistical evaluation, the data were compared using t-test. A p-value of less than 0.05 was considered statistically significant. Data are represented as mean ± standard error of the mean (SEM) calculated from three independent experiments, each performed in duplicate. The linear-quadratic (LQ) model was fitted using the least squares method, and the goodness of fit was evaluated based on the coefficient of determination ${\text{R}}^{2}$.

## Results

### Characterization of sEVs

**Fig 2:**
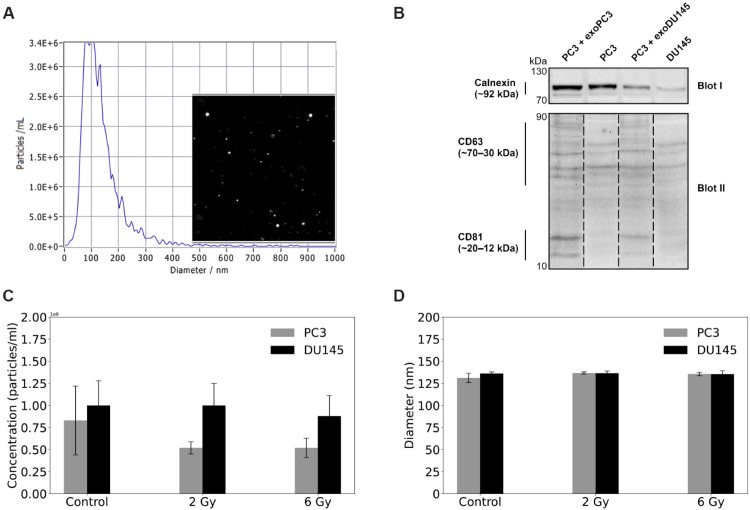
Characterization of small extracellular vesicles (sEVs) derived from two prostate cancer cell lines: PC3 and DU145. A: Representative concentration and size distribution plot of PC3-derived sEVs measured by NTA and particle visualization based on Brownian motions. B: Representative immunoblot of sEVs markers from two separate blots: Calnexin (blot I), CD63 and CD81 (blot II) in PC3-derived sEVs (PC3 + exoPC3) and DU145-derived sEVs (PC3 + exoDU145) harvested after 42 h of co-incubation with PC3 cells and for cell lysates (PC3 and DU145). C: Particle concentration in response to ionizing radiation (2 Gy and 6 Gy) and control samples (non-irradiated, 0 Gy). Results were obtained using NTA. D: Particle diameter under irradiated and control conditions. Results were obtained using NTA. All data represent three independent experiments in two biological replicates each and are presented as means ± standard error of the mean (SEM).

sEVs were isolated from supernatants of human prostate cancer cell lines PC3 and DU145 using size-exclusion chromatography (SEC). In our previous studies we confirmed by atomic force microscopy (AFM) imaging, that the recovered exosomes are morphologically intact, aggregate-free [33]. The size distribution of vesicles released from irradiated cells (2 Gy and 6 Gy) and control cells (non-irradiated, 0 Gy) was analyzed by nanoparticle tracking analysis (NTA), which revealed an mean particle diameters ranging from 130 to 137 nm (Fig 2A). The vesicles were characterized by the presence of typical exosome biomarkers, including CD63 and CD81, which were confirmed by Western blot analysis (Fig 2B). Based on this confirmation the small EVs are referred to as exosomes from this point forward. Exosome characterization was performed for both cell lysates and PC3 cells incubated with exosomes derived from PC3 (PC3 + exoPC3) and DU145 (PC3 + exoDU145) cells. Densitometric analysis of the signal intensity for CD63 and CD81 revealed that PC3 + exoPC3 contained, on average, 32% more CD63 and 73% more CD81 than PC3 cells. A similar trend was observed for PC3 + exoDU145, 65% more CD63 and 61% more CD81 compared to DU145 cells. In summary, the cell lysates exhibited lower levels of CD63 and CD81 than the samples incubated with exosomes. In our study, although not all canonical markers were uniformly detected, we consistently observed a stronger CD63 signal, particularly in cells co-incubated with sEVs. Notably, there was a visible and reproducible increase in CD63 staining in recipient cells exposed to sEVs compared to untreated controls. This finding supports the successful transfer or uptake of CD63-positive sEVs and reinforces the functional relevance of the vesicles used in our experiments. The concentration of exosomes derived from DU145 cells exposed to X-rays radiation, as determined by NTA, was greater than those isolated from PC3 cells, with irradiated DU145 samples yielding approximately twice the concentration. No statistically significant difference in exosome numbers was observed between the two prostate cancer cell lines (Fig 2C). By NTA, the mean particle diameters were (131.2 ± 5.4) nm for non-irradiated cells to (136.7 ± 1.3) nm and (135.6 ± 1.9) nm for irradiated PC3 cells (2 Gy and 6 Gy, respectively) and (136.1 ± 1.8) nm for non-irradiated cells to (136.5 ± 2.5) nm and (135.4 ± 3.8) nm for irradiated DU145 cells (2 Gy and 6 Gy, respectively), which is consistent with the expected size of exosomes (Fig 2D). No correlation was observed between the exosomes size and the dose delivered to the cells. Moreover, no significant differences were found in the average diameters of the exosomes between radiation doses (*p*>0.05).

### Cell survival

**Fig 3:**
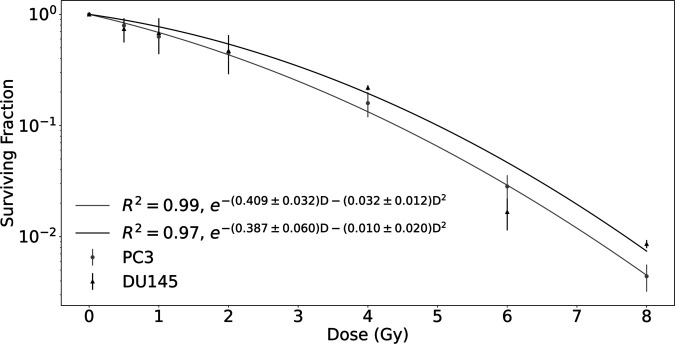
Survival curves for the different prostate cancer cell lines, PC3 and DU145, obtained after exposure to different doses of X-rays. Data were fitted to the linear-quadratic model. Points represent the mean of at least three independent experiments, each with three biological replicates, and are presented with SEM.

To determine the cellular response to X-ray radiation, a clonogenic survival assay was performed. The surviving fraction of PC3 cells and DU145 cells as a function of the absorbed dose are presented in Fig 3. A strong cellular response to X-ray irradiation was observed, as demonstrated by a surviving fraction at 8 Gy (*SF*8) of (0.0044 ± 0.0012) for PC3 cells and (0.00860 ± 0.00070) for DU145 cells. DU145 cells exhibit higher radioresistance than PC3 cells, as indicated by the higher value of *SF* in DU145 cells exposed to X-ray radiation. Moreover, for PC3 cells, the fitted parameters were *α* = (0.409 ± 0.032)Gy^−1^ and *β* = (0.032 ± 0.012)Gy^−2^, yielding an *α*/*β* ratio of approximately 12.8 Gy. For DU145 cells, the values were *α* = (0.387 ± 0.060)Gy^−1^ and *β* = (0.010 ± 0.020)Gy^−2^, resulting in an *α*/*β* ratio of about 38.7 Gy. Although both cell lines exhibit relatively high *α*/*β* ratio – indicative of a more linear radiation response and lower sensitivity to dose fractionation – the higher value for DU145 line suggests higher intrinsic radioresistance relative to PC3 cells. This interpretation is consistent with current radiobiological understanding of *α*/*β* ratios, as discussed by Bodgi et al. [34] in their comprehensive review of radiation action models.

### Apoptosis

**Fig 4:**
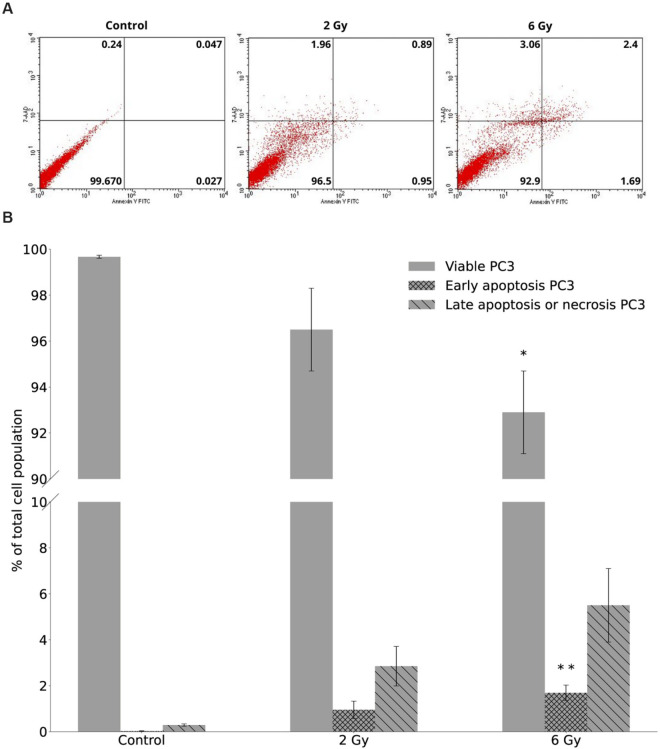
The dose response on viable cells, early and late apoptosis or necrosis in PC3 cells, as detected 96 hours after X-ray irradiation by flow cytometry. Data were averaged from three independent experiments, each performed in two replicants for 0 Gy (control), 2 Gy, and 6 Gy. A: Diagrams of FITC-Annexin V/7-ADD flow cytometry. The lower left quadrant represents viable cells (Annexin V-FITC negative and 7-ADD negative staining). The lower right quadrant represents early apoptotic cells (Annexin V-FITC positive and 7-ADD negative staining). The upper right and upper left quadrants contain late-stage apoptotic cells or necrotic cells (Annexin V-FITC positive and 7-ADD positive and Annexin V-FITC negative and 7-ADD positive staining, respectively). B: Data are presented as % of viable cells, cells at an early stage of apoptosis and as % of cells at late-stage apoptosis or necrotic cells. Error bars represent standard error of the mean, ^⋆⋆^ *p* ≤0.01, ^⋆^ *p* ≤ 0.05, is calculated as compared to control and marked as ^⋆^.

**Fig 5:**
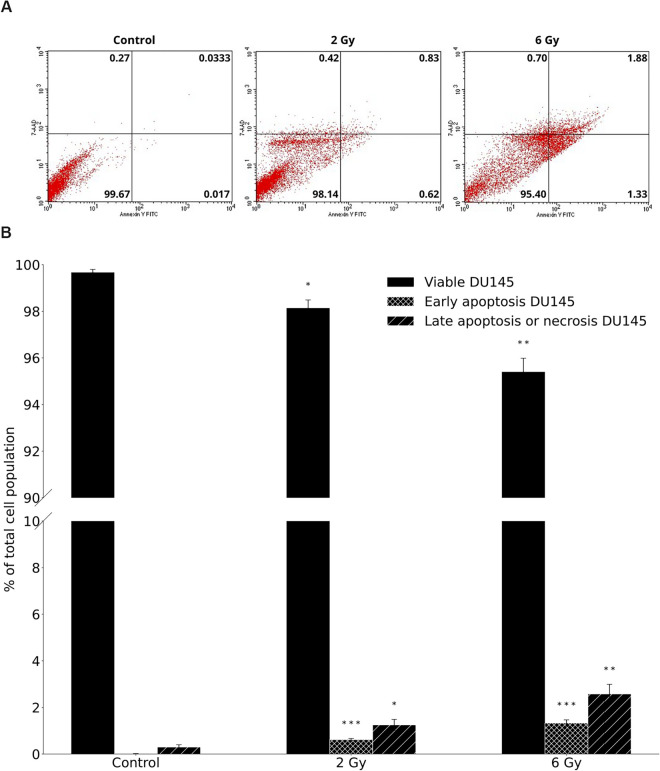
The dose response on viable cells, early and late apoptosis or necrosis in DU145 cells, as detected 96 hours after X-ray irradiation by flow cytometry. Data were averaged from three independent experiments, each performed in two replicants for 0 Gy (control), 2 Gy, and 6 Gy. A: Diagrams of FITC-Annexin V/7-ADD flow cytometry. The lower left quadrant represents viable cells (Annexin V-FITC negative and 7-ADD negative staining). The lower right quadrant represents early apoptotic cells (Annexin V-FITC positive and 7-ADD negative staining). The upper right and upper left quadrants contain late-stage apoptotic cells or necrotic cells (Annexin V-FITC positive and 7-ADD positive and Annexin V-FITC negative and 7-ADD positive staining, respectively). B: Data are presented as % of viable cells, cells at an early stage of apoptosis and as % of cells at late-stage apoptosis or necrotic cells. Error bars represent SEM, ^⋆⋆⋆^ *p* ≤ 0.001, ^⋆⋆^ *p* ≤0.01, ^⋆^ *p* ≤ 0.05, is calculated as compared to control and marked as ^⋆^.

To interpret the fundamental mechanism of cell death induction, the effect of X-rays on viable cells, early and late stage apoptosis or necrosis was studied using Annexin V-FITC/7-AAD flow cytometry analysis. The average numbers of viable cells, early and late apoptotic or necrotic cells are shown for PC3 cells in Fig 4B, based on diagrams (see Fig 4A) of flow cytometry. Corresponding data for the DU145 cell line are presented in Fig 5A and 5B. Annexin V-FITC/7-AAD staining revealed a dose-dependent decrease in the proportions of viable cells and increase in the proportions of early apoptotic and late apoptotic or necrotic cells in both PC3 and DU145 cell lines following X-ray exposure.

This effect was particularly evident in PC3 cells, where a significant decrease in the number of viable cells was observed compared to the control. The statistically significant difference was noted only at 6 Gy (*p* ≤ 0.05). Simultaneously, the numbers of apoptotic and necrotic PC3 cells increased with radiation dose. The level of statistical significance for the number of dead PC3 cells for control and 6 Gy irradiated samples was observed only for early apoptosis (*p* ≤ 0.01). Late apoptotic or necrotic cells also followed this trend, although statistical significance was not noticed.

The similar pattern was observed for DU145 cells. The statistical significance for viable cells was observed at 2 Gy (*p* ≤ 0.05) and 6 Gy (*p* ≤ 0.01) for DU145 cells compared to control. The level of statistical significance for the number of dead DU145 cells for control and 6 Gy irradiated samples was observed for early apoptosis (*p* ≤ 0.001) and late apoptosis or necrosis (*p* ≤ 0.01). For DU145 cells, we also observed significant differences between non-irradiated cells and the radiation dose equal 2 Gy (*p* ≤ 0.001 and *p* ≤ 0.05 for early and late apoptosis, respectively).

The difference in the number of viable cells was higher for PC3 (96.5 ± 1.8 and 92.9 ± 1.8) than for DU145 (98.14 ± 0.62 and 95.40 ± 1.33) cells for 2 Gy and 6 Gy, respectively. The numbers of early apoptotic cells were higher for PC3 (0.95 ± 0.38 after 2 Gy and 1.69 ± 0.34 after 6 Gy) compared to DU145 cells (0.62 ± 0.05 and 1.33 ± 0.14 for 2 Gy and 6 Gy, respectively). The similar pattern was observed for late apoptotic or necrotic cells: following 2 Gy irradiation, the values were 2.85 ± 0.86 for PC3 cells and 1.25 ± 0.24 for DU145 cells, while after 6 Gy of X-rays, the values increased to 5.5 ± 1.6 and 2.58 ± 0.41 for PC3 and DU145 cells, respectively. Furthermore, PC3 cells exhibited higher radiosensitivity than DU145 cells, as supported by the increased number of late apoptotic or necrotic cells (approximately twofold higher) after X-rays irradiation.

### Apoptosis bystander effect

**Fig 6:**
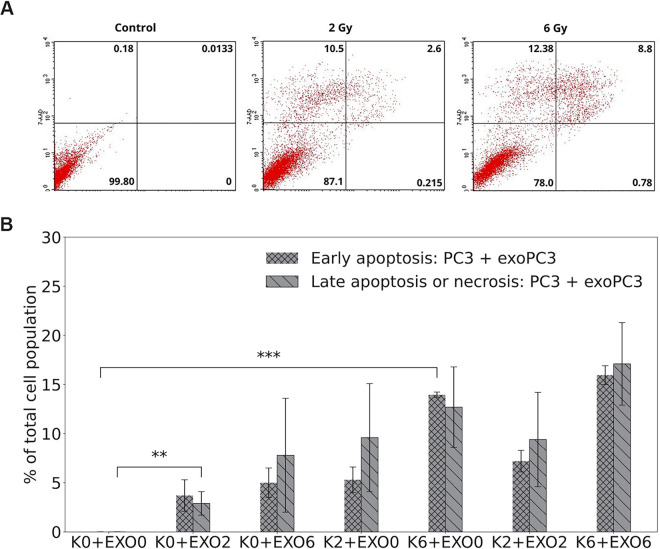
The dose-effect of X-ray radiation on early and late apoptosis or necrosis in exosome-treated cells (PC3 cells and exosomes released by PC3) as detected 96 hours after irradiation by flow cytometry. Data are averaged from 3 independent experiments, each in two replicants performed for: control PC3 cells and exosomes released by control PC3 cells (K0+EXO0), control PC3 cells and exosomes released by irradiated PC3 cells (K0+EXO2 and K0+EXO6), irradiated PC3 cells and exosomes released by control PC3 cells (K2+EXO0 and K6+EXO0), and irradiated PC3 cells and exosomes released by irradiated PC3 cells (K2+EXO2 and K6+EXO6). A: Diagrams of FITC-Annexin V/7-ADD flow cytometry. The lower left quadrant represents viable cells (Annexin V-FITC negative and 7-ADD negative staining). The lower right quadrant represents early apoptotic cells (Annexin V-FITC positive and 7-ADD negative staining). The upper right and upper left quadrants contain late-stage apoptotic cells or necrotic cells (Annexin V-FITC positive and 7-ADD positive and Annexin V-FITC negative and 7-ADD positive staining, respectively). The dose-effect of X-ray radiation on viable cells, early and late apoptosis or necrosis in PC3 cells as detected 96 hours after irradiation by flow cytometry and averaged from 3 independent experiments, each in two replicants performed for 0 Gy (control), 2 Gy, and 6 Gy. Cells were cultured in Dulbecco’s modified Eagle medium (DMEM-F12) supplemented with 5% Fetal Bovine Serum, exosome-depleted and 2.0% penicillin-streptomycin. B: Data are presented as % of cells at an early stage of apoptosis and as % of cells at late-stage apoptosis or necrotic cells. Error bars represent SEM, ^⋆⋆⋆^ *p* ≤ 0.001, ^⋆⋆^ *p* ≤ 0.01 is calculated as compared to appropriate control and marked as ^⋆^.

**Fig 7:**
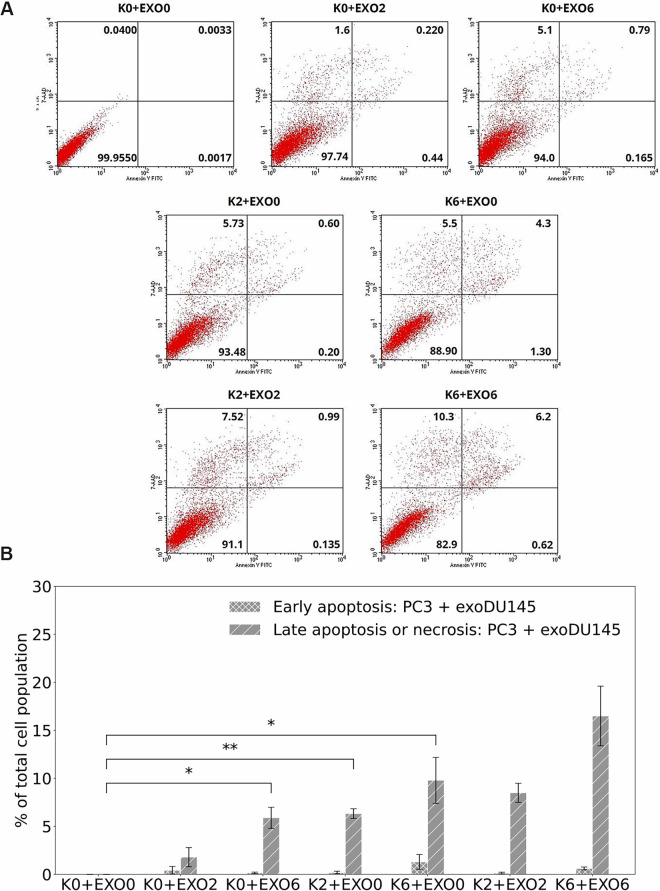
The dose-effect of X-ray radiation on viable cells, early and late apoptosis or necrosis in PC3 cells co-incubated with exosomes isolated from DU145 cells, as detected 96 hours after irradiation by flow cytometry. Data were averaged from 3 independent experiments, each in two replicants performed for: control PC3 cells and exosomes released by control DU145 cells (K0+EXO0), control PC3 cells and exosomes released by irradiated DU145 cells (K0+EXO2 and K0+EXO6), irradiated PC3 cells and exosomes released by control DU145 cells (K2+EXO0 and K6+EXO0), and irradiated PC3 cells and exosomes released by irradiated DU145 cells (K2+EXO2 and K6+EXO6). A: Diagrams of FITC-Annexin V/7-ADD flow cytometry. The lower left quadrant represents viable cells (Annexin V-FITC negative and 7-ADD negative staining). The lower right quadrant represents early apoptotic cells (Annexin V-FITC positive and 7-ADD negative staining). The upper right and upper left quadrants contain late-stage apoptotic cells or necrotic cells (Annexin V-FITC positive and 7-ADD positive and Annexin V-FITC negative and 7-ADD positive staining, respectively). The dose response on viable cells, early and late apoptosis, and necrosis in PC3 cells co-incubated with exosomes isolated from DU145 cells, as detected 96 hours after irradiation using flow cytometry. Results represent the mean values from three independent experiments, each conducted in duplicate, with radiation doses of 0 Gy (control), 2 Gy, and 6 Gy applied either directly to the cells or indirectly via exosome-mediated exposure. Cells were cultured in Dulbecco’s modified Eagle medium (DMEM-F12) supplemented with 5% Fetal Bovine Serum, exosome-depleted and 2.0% penicillin-streptomycin. B: Data are presented as % of cells at an early stage of apoptosis and as % of cells at late-stage apoptosis or necrotic cells. Error bars represent SEM, ^⋆⋆^ *p* ≤ 0.01, and ^⋆^ *p* ≤ 0.05, is calculated as compared to appropriate control and marked as ^⋆^.

To investigate the potential activation of RIBE in exosome-stimulated cells, we examined apoptotic cell death. The averaged number of viable cells, as well as those undergoing early and late apoptosis or necrosis, were quantified based on diagrams of FITC-Annexin V/7-ADD flow cytometry. The results were obtained for two scenarios of PC3 co-incubation: PC3 cells co-incubated with exosomes released by PC3 (see Fig 6) and PC3 cells co-incubated with exosomes released by DU145 cells (see Fig 7).

We found that the difference in the number of viable cells and late apoptotic or necrotic cells was higher for irradiated PC3 cells supplemented with 5% Gibco Exosome-Depleted FBS (viable: 87.1 ± 2.4 for 2 Gy, 78.0 ± 4.2 for 6 Gy, and late apoptotic or necrotic: 13.1 ± 1.8 for 2 Gy, 21.2 ± 3.8 for 6 Gy; as shown in Fig 6A) compared to PC3 cells supplemented with 10% FBS (viable: 96.5 ± 1.8 for 2 Gy, 92.9 ± 1.8 for 6 Gy, and late apoptotic or necrotic: 2.85 ± 0.86 for 2 Gy, 5.5 ± 1.6 for 6 Gy; as shown in Fig 4A).

For irradiated PC3 cells stimulated by exosomes released from non-irradiated PC3 cells (K2+EXO0 and K6+EXO0), we observed an increased number of early and late apoptotic or necrotic cells compared to non-irradiated cells stimulated by exosomes released from non-irradiated PC3 cells (K0+EXO0). The statistical significance for early apoptosis was observed for K6+EXO0 (*p* ≤ 0.001) compared to the control (K0+EXO0).

Subsequently, we compared non-irradiated PC3 cells treated with exosomes isolated from irradiated PC3 cells (K0+EXO2 and K0+EXO6) to K0+EXO0 samples. We also observed the greater number of early apoptotic and late apoptotic or necrotic cells for K0+EXO2 and K0+EXO6 samples compared to the control (K0+EXO0). For K0+EXO2 samples, a statistically significant increase in the late apoptosis or necrosis was observed (*p* ≤ 0.01) compared to K0+EXO0 samples.

We also compared irradiated PC3 cells treated with exosomes isolated from non-irradiated PC3 cells (K2+EXO0 and K6+EXO0) to irradiated PC3 cells treated with exosomes isolated from irradiated PC3 cells (K2+EXO2 and K6+EXO6). For these samples no statistically significant differences were observed. Regarding the number of apoptotic and necrotic cells, a trend of increasing numbers of cells in early and late stages of apoptosis or necrosis depending on the dose, was noted. Since cells in this group were consistently irradiated, this trend was evident when comparing exosomes isolated from control cells to those isolated from irradiated cells.

In the second configuration (PC3 + exoDU145), PC3 cells were stimulated by exosomes released from DU145 cells. For the first group of samples, where PC3 cells were irradiated and exosomes were isolated from non-irradiated DU145 cells (K2+EXO0 and K6+EXO0), we also observed an increased number of early and late apoptotic or necrotic cells compared to K0+EXO0 samples. The difference in number of early and late apoptotic or necrotic cells was also higher for K6+EXO0 than for K2+EXO0. The statistical significance for late apoptosis or necrosis was observed for K2+EXO0 (*p* ≤ 0.01) and K6+EXO0 (*p* ≤ 0.05) compared to the control (K0+EXO0).

For the second group of samples, we compared non-irradiated PC3 cells treated with exosomes isolated from irradiated DU145 cells (K0+EXO2 and K0+EXO6) to control samples (K0+EXO0). We also observed the greater number of early apoptotic and late apoptotic or necrotic cells, with an increasing dose delivered to the cells from which the exosomes were isolated. For K0+EXO6 samples, a statistically significant increase in the late apoptosis or necrosis was observed (*p* ≤ 0.05) compared to K0+EXO0 samples.

In the third group of samples, we compared irradiated PC3 cells treated with exosomes isolated from non-irradiated DU145 cells (K2+EXO0 and K6+EXO0) to irradiated PC3 cells treated with exosomes isolated from irradiated DU145 cells (K2+EXO2 and K6+EXO6). Similarly to the first configuration, no statistically significant differences were observed. However, a trend was noted in which the number of apoptotic and necrotic cells increased with dose. Since all cells in this group were irradiated, this trend was specifically observed when comparing exosomes isolated from control cells and those isolated from irradiated DU145 cells.

In summary, in the first configuration (PC3 + exoPC3), the number of early and late apoptotic or necrotic cells was higher for K6+EXO0 (13.96 ± 0.27 and 12.7 ± 4.1, respectively) than for K2+EXO0 (5.3 ± 1.3 and 9.6 ± 5.5, respectively). In the second configuration (PC3 + exoDU145), the number of early and late apoptotic or necrotic cells was also higher for K6+EXO0 (1.30 ± 0.77 and 9.8 ± 2.4, respectively) than for K2+EXO0 (0.20 ± 0.13 and 6.33 ± 0.50, respectively). The difference in the number of early and late apoptotic or necrotic cells was higher for PC3 + exoPC3 than for PC3 + exoDU145 configuration.

We also observed a greater number of early apoptotic cells in the PC3 + exoPC3 configuration, with values of 3.7 ± 1.6 for K0+EXO2 and 5.0 ± 1.5 for K0+EXO6, as well as in the PC3 + exoDU145 configuration, where the values were 0.43 ± 0.40 for K0+EXO2 and 0.165 ± 0.072 for K0+EXO6. Similarly, the number of late apoptotic or necrotic cells was also greater, with PC3 + exoPC3 showing values of 2.9 ± 1.2 for K0+EXO2 and 7.8 ± 5.8 for K0+EXO6, while PC3 + exoDU145 exhibited 1.8 ± 1.0 for K0+EXO2 and 5.9 ± 1.1 for K0+EXO6. These results indicate an increase in apoptotic and necrotic cell populations compared to non-irradiated cells stimulated by exosomes released from non-irradiated cells (K0+EXO0).

Based on the data (Figs 6 and 7), statistically significant differences in early apoptosis between the PC3 + exoPC3 and PC3 + exoDU145 groups were observed for the following conditions: K6+EXO0 (*p* ≤ 0.001), K2+EXO2 (*p* ≤ 0.05), and K6+EXO6 (*p* ≤ 0.001).

**Fig 8:**
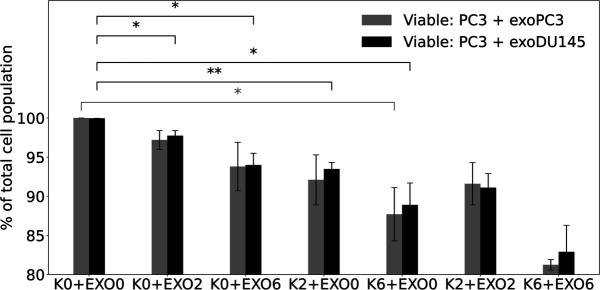
The dose-effect of X-ray radiation on viable cells in exosome-treated cells for two configuration: PC3 cells and exosomes released by PC3 (PC3 + exoPC3) and PC3 cells and exosomes released by DU145 (PC3 + exoDU145) as detected 96 hours after irradiation by flow cytometry. Data are averaged from 3 independent experiments, each in two replicants performed for: control cells and exosomes released by control cells (K0+EXO0), control cells and exosomes released by irradiated cells (K0+EXO2 and K0+EXO6), irradiated cells and exosomes released by control cells (K2+EXO0 and K6+EXO0), and irradiated cells and exosomes released by irradiated cells (K2+EXO2 and K6+EXO6). Data are presented as % of viable cells. Error bars represent SEM, ^⋆⋆^ *p* ≤0.01 and ^⋆^ *p* ≤ 0.05, is calculated as compared to appropriate control and marked as ^⋆^.

The difference in the number of viable cells was higher for PC3 + exoPC3 than for PC3 + exoDU145 (Fig 8). For the first group, K2+EXO0 and K6+EXO0 compared to K0+EXO0, the level of statistical significance for the number of viable cells was greater for K2+EXO0 (*p* ≤ 0.01) than for K6+EXO0 (*p* ≤ 0.05) for PC3 + exoDU145 samples, while for PC3 + exoPC3 samples, significance was noted only at K6+EXO0 (*p* ≤ 0.05) compared to K0+EXO0. For the K0+EXO2 and K0+EXO6 compared to K0+EXO0, the statistical significance for viable cells was observed at K0+EXO2 and K0+EXO6 (*p* ≤ 0.05) only for PC3 + exoDU145 samples. A statistically significant difference in viable cell numbers between the PC3 + exoPC3 and PC3 + exoDU145 groups for the K0+EXO0 condition (*p* ≤ 0.01) was determined based on the data shown in Fig 8. The obtained results confirmed our previous hypothesis that DU145 cells are more radioresistant than PC3 cells and that exosomes isolated from these cells could play the role in the resistance process of human prostate cancer cell lines to ionizing radiation.

### *γ*H2AX Immunostaining

**Fig 9:**
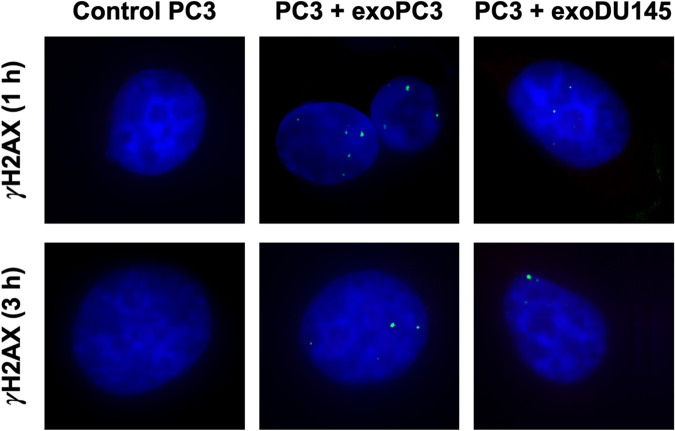
Microscopic images of PC3 nuclei showing the induction of *γ*H2AX foci by exosomes secreted from irradiated PC3 and DU145 cells. Visualization of *γ*H2AX foci in non-irradiated PC3 cells co-incubated for 1 and 3 hours with exosomes released by PC3 and DU145 cells exposed to 2 Gy of X-rays (PC3 + exoPC3 and PC3 + exoDU145). Non-irradiated cells (Control PC3) were used as controls.

To investigate whether a hypothetical RIBE could be activated in recipient cells by exosomes, the presence of the *γ*H2AX foci in the nuclei of recipient cells was analysed. Fig 9 presents microscopic images of *γ*H2AX foci in the nuclei of non-irradiated PC3 cells, co-incubated for 1 h and 3 h with exosomes isolated from irradiated PC3 and DU145 cells. The average number of *γ*H2AX foci per nucleus in control PC3 cells was 0.914 ± 0.076 after 1 hour and 0.890 ± 0.081 after 3 hours. For the samples co-incubated with exosomes (PC3 + exoPC3 and PC3 + exoDU145), the corresponding values were 4.37 ± 0.73 and 3.87 ± 0.89 after 1 hour, and 3.22 ± 0.70 and 2.43 ± 0.21 after 3 hours of incubation, respectively. Statistically significant differences (*p* ≤ 0.05) in the average number of DNA repair foci per nucleus were observed between control PC3 cells and those co-incubated for 1 hour and 3 hours with exosomes isolated from irradiated PC3 and DU145 cells. Although no statistically significant difference was found between the PC3 + exoPC3 and PC3 + exoDU145 groups, a trend toward a higher number of *γ*H2AX foci was observed in the PC3 + exoPC3 group, suggesting a more pronounced bystander DNA damage response. This finding aligns with the notion that DU145 cells exhibit a more radioresistant phenotype compared to PC3 cells. We demonstrated that the numbers of *γ*H2AX foci in these nuclei were greater than in the nuclei of control (non-irradiated PC3 cells). This suggests that exosomes released by irradiated cells can induce RIBE in non-irradiated cells. Higher levels of *γ*H2AX foci were observed in the PC3 + exoPC3 configuration compared to the PC3 + exoDU145 configuration, further indicating that DU145 cells are more resistant to radiation than PC3 cells.

## Discussion

In the present study, we used two human prostate cancer cell lines, PC3 and DU145, to investigate the effects of low LET X-ray radiation, by assessing their radiosensitivity through cell survival and apoptosis assays. Based on cell survival curves we confirmed that DU145 cells exhibit a greater magnitude of radioresistance than PC3 cells. This observation aligned with the results of a previous study [25,35], where human prostate cancer cell lines were irradiated using a Siemens Stabilipan 250 keV X-ray machine and ^60^Co gamma rays, respectively. Parameters obtained from the fitted curves and *SF*2 analyzed with 3*σ* criterion can be considered with the data from van Oorschot et al. [35]. We observed the difference in the number of apoptotic and viable cells between PC3 and DU145, with PC3 showing an approximately twofold increase in late apoptotic or necrotic cells compared to DU145, indicating their higher radiosensitivity. Zhao et al. [36] demonstrated an increase in the percentage of apoptotic cells following X-rays radiation as a function of time. In the work by Zhao et al. [36], the dose-response of apoptosis was investigated by harvesting HeLa cells at 48 and 72 hours after irradiation, with 72 hours being consistent with our experimental setup. Furthermore, based on our previously published data on apoptosis in PC3 and DU145 cells following exposure to densely ionizing alpha particles [37], we observed that high LET radiation induces significantly higher levels of apoptosis compared to the low LET X-ray radiation investigated in the present study.

In the present work, we investigated whether irradiation-induced stress affects the concentration and size of exosomes isolated from human prostate cancer cell lines. Using NTA, we observed that due to large SEM values, there was no statistically significant difference in the number or size of exosomes released by PC3 and DU145 with increasing radiation doses. In [37], we reported a decrease in exosome secretion as a function of radiation dose delivered by alpha particles. This finding aligns with a study [38], where human fetal lung fibroblast HF19 cells were irradiated using a ^238^Pu alpha-particle source. That study also reported a significant increase in exosome concentration following X-rays irradiation and non-significant changes in exosome size across all irradiated groups compared to control (non-irradiated). In other studies on U87 glioma cells [39] the Zetasizer analysis demonstrated that size of exosomes was not affected by radiation exposure, which is consistent with our results. In our experiment, we observed that the concentration of exosomes derived from DU145 cells exposed to X-ray radiation was significantly higher than that from irradiated PC3 cells, with DU145 samples yielding approximately twice the amount. Several phenotypic and mechanistic factors may account for this difference. DU145 cells are known to be more radioresistant than PC3 cells [25]. Emerging evidence suggests that radioresistant cancer cells may secrete more exosomes in response to stress [40,41]. Exosomes can modulate the tumor microenvironment, facilitate the removal of damaged cellular components, and transfer molecules to neighboring cells. Therefore, increased exosome release in DU145 may represent a survival strategy involving intercellular communication. Phenotypic differences between the two cell lines may also contribute. DU145 cells are more differentiated and exhibit a predominantly epithelial phenotype, whereas PC3 cells are less differentiated and display features associated with epithelial-mesenchymal transition [42,43]. Some studies suggest that epithelial-type cancer cells may produce more exosomes than mesenchymal-like cells, although this relationship is complex and context-dependent [44,45]. Moreover, DU145 and PC3 cells differ in the activation of major signaling pathways, including p53 (which regulates the transcription of genes involved in vesicle formation and export), mTOR (mTORC1 influences exosome release through its role in autophagy and endosomal dynamics), and others such as PI3K/AKT and MAPK/ERK [23,46]. DU145 cells may activate stress-response pathways more effectively, promoting exosomal secretion as a compensatory mechanism. Finally, technical variables such as differences in proliferation rates, cell size, or exosome biophysical properties may influence isolation yields. Further studies are required to elucidate the molecular determinants underlying exosome production in these cell lines, including comparative analyses of exosomal cargo, expression of biogenesis-related genes, and the effects of radiation on stress-related signaling pathways.

Research on exosomes is becoming increasingly important in the field of radiobiology. Radiation impacts not only the directly exposed cells but also nearby non-irradiated cells, a response described as radiation-induced bystander effects (RIBE), which is initiates by molecular communication [47,48]. Therefore, in the present study, we first investigated the cellular responses to 2 Gy X-ray (a radiotherapeutic dose of IR) and 6 Gy X-ray (as a palliative RT treatment) [49] for human prostate cancer cell lines, in terms of exosome profile including exosome concentration and size. Subsequently, we explored the role of exosomes as mediators of radiation-induced damage was investigated by measuring cell death apoptosis, *γ*H2AX immunostaining and immunoblotting in exosome-exposed PC3 cells. The exosomes isolated from DU145 cells (radioresistance) were uptaken by recipient cells PC3 (radiosensitivity) and were shown that exosomes secreted from radioresistance cancer cell lines influence on ionizing radiation effects, weakening it. The difference in the number of viable cells was higher for PC3 + exoPC3 than for PC3 + exoDU145. For non-irradiated PC3 cells stimulated by exosomes released from irradiated PC3 cells, we observed the greater number of early apoptotic and late apoptotic or necrotic cells compared to non-irradiated cells stimulated by exosomes released from non-irradiated PC3 cells. The similar trend was observed for non-irradiated PC3 cells stimulated by exosomes released from irradiated DU145 cells compared to non-irradiated PC3 cells stimulated by exosomes released from non-irradiated DU145 cells, but the number of cell death was lower in this configuration. We demonstrated that the numbers of *γ*H2AX foci in the nuclei of control PC3 cells co-incubation with exosomes isolated from irradiated PC3 and DU145 cells were greater than in the nuclei of control (non-irradiated PC3 cells). This suggests that exosomes released by irradiated cells induced RIBE in non-irradiated cells. In work [50], naive FaDu head and neck cancer cells were stimulated with exosomes released by irradiated or mock-irradiated cells. Smolarz et al. showed that the levels of *γ*H2AX foci in the nuclei of cells directly exposed to a 2 Gy dose (1 hour post-irradiation) and in the nuclei of Ex_2Gy-stimulated cells (after 1 hour of incubation) were similarly high. In contrast, the generally low levels of *γ*H2AX foci in Ex_0Gy-stimulated cells were comparable to those in naive, untreated control cells.

This study, which explores various aspects, including the release and uptake of exosomes by human prostate cancer cell lines, confirms that RIBE can be triggered in exosome-stimulated cells. Consistent with this, recent research has shown that exosomes released by irradiated cells can induce ionizing radiation-related effects in unirradiated recipient cells [51,52]. Moreover, exosomes have been implicated in RIBE in cancer cells and in the development of resistance to radiotherapy [53–55]. Mechanistically, ionizing radiation alters exosomal secretion patterns and the composition of exosomal cargo in target cells, which, as demonstrated in multiple in vitro studies, can induce RIBE in bystander cells [56]. Exosomes can transfer specific microRNAs (miRNAs) from irradiated cells to non-irradiated bystander cells, influencing gene expression and cellular responses like DNA damage, apoptosis, or inflammation. miR-21 is shown to be upregulated in exosomes from irradiated cells and transferred to bystander cells, promoting DNA damage and reactive oxygen species production [15]. miR-1246 is involved in targeting DNA damage repair pathways, affecting recipient cell susceptibility to radiation [57]. Exosomes can carry double-stranded DNA (dsDNA) fragments or mitochondrial DNA from irradiated cells, which can act as damage-associated molecular patterns, activating DNA sensing pathways in bystander cells. Exosomal DNA activates the cGAS-STING pathway in bystander cells, leading to the induction of type I interferons and inflammatory signaling [16]. Exosomes can carry surface ligands, such as heat shock proteins (HSP70/90) or death ligands (FasL, TRAIL), which interact with receptors on bystander cells, initiating signaling cascades associated with stress responses or apoptosis [17,58].

The role of ionizing radiation in exosome signaling is a critical mechanism for understanding key regulators of radioresistance, which could lead to improved clinical safety and radiotherapy efficiency. To enhance our understanding of the influence of exosome-induced radioresistance, further research is needed to elucidate the mechanisms underlying exosome-mediated radiation-induced effects in prostate cancer cells. Moreover, exploring how exosomes and their contents influence the tumor microenvironment could shed light on pathways associated with radiation resistance and aid in developing more effective exosome-based strategies for tumour radiotherapy [59].

## Conclusion

In conclusion, this study highlights the potential role of exosomes in mediating radiation-induced bystander effects in cancer cells. The findings suggest that exosomes released from irradiated cells can influence the behavior of non-irradiated cells, contributing to increased cell death and DNA damage. These results underscore the importance of exosome-mediated communication in the cellular response to ionizing radiation and its potential impact on cancer treatment outcomes. Further investigation into the mechanisms of exosome signaling could provide valuable insights for improving radiotherapy strategies and addressing radiation resistance in tumors.
